# Teamwork Makes the Dream Work: Who Should Be Managing Athletes on Matters Related to Sleep?

**DOI:** 10.1007/s40279-025-02282-7

**Published:** 2025-07-25

**Authors:** Matthew W. Driller, Shona L. Halson, Cheri D. Mah, Haresh Suppiah, Michele Lastella, Dean J. Miller, Matthew B. Cooke, Ebonie Rio, Alan St. Clair Gibson, Amy M. Bender, Rachel Harris, Stuart Armstrong, Gary Slater, Michael A. Grandner

**Affiliations:** 1 Sport, Performance, and Nutrition Research Group, School of Allied Health, Human Services, and Sport, La Trobe University, Melbourne, Australia; 2 School of Behavioural and Health Sciences, Australian Catholic University, Banyo, Brisbane, Australia; 3 Department of Psychiatry and Behavioral Sciences, Stanford Sleep Medicine Center, Stanford University, Stanford, USA; 4 Appleton Institute, Central Queensland University, Adelaide, SA Australia; 5 School of Health, Medical and Applied Sciences, Central Queensland University, Rockhampton, QLD Australia; 6 The Victorian Institute of Sport, Melbourne, Australia; 7 La Trobe Sport and Exercise Medicine Research Centre, La Trobe University, Melbourne, Australia; 8 Institute of Applied Health Sciences, School of Medical Studies and Nutrition, University of Aberdeen, Aberdeen, UK; 9 Faculty of Kinesiology, University of Calgary, Calgary, AB Canada; 10 Female Performance and Health Initiative, Australian Institute of Sport, Canberra, ACT Australia; 11 Perth Orthopaedic and Sports Medicine Research Institute, West Perth, WA Australia; 12 Angelsea Sports Medicine, Hamilton, New Zealand; 13 School of Health, University of the Sunshine Coast, Maroochydore, DC Australia; 14 Australian Institute of Sport, Canberra, ACT Australia; 15 Sleep and Health Research Program, Department of Psychiatry, University of Arizona College of Medicine - Tucson, Tucson, AZ USA

## Abstract

Optimising sleep health is essential for athlete recovery and performance, but responsibility for managing sleep in high-performance sports is often unclear. Although performance support teams include diverse professionals such as coaches, sport scientists, physiotherapists, sports dietitians, psychologists, and physicians, guidance may be fragmented and inconsistent across training environments and competition schedules. This paper proposes a collaborative multidisciplinary model in which sleep specialists may integrate with existing support staff to deliver unified, evidence-based sleep strategies throughout all phases of athlete preparation. By fostering open communication channels, aligning screening protocols, and coordinating interventions, this model ensures consistent messaging and implementation of sleep initiatives. We also address sleep monitoring via wearable technologies, highlighting device performance and data confidentiality considerations to ensure accurate and ethical use of athlete sleep metrics. Emphasis is placed on creating organisation-wide policies that recognise sleep as an important component to health and performance. Adopting this integrated approach to sleep may enhance overall physical and mental health, reduce injury risk, improve recovery, and ultimately, enhance athletic performance.

## Key Points

**Table:** 

A multidisciplinary team of professionals is essential to deliver consistent, evidence-based sleep management for athletes.
Integrating sleep specialists into the support team (where possible) ensures that education, screening, monitoring, and interventions are tailored and coordinated, with careful attention to device performance and athlete data confidentiality.
Adopting a unified approach to athlete sleep can enhance recovery, performance, and overall well-being.

## Introduction

In the competitive world of elite sports, enhancement of health and wellness can make the difference between winning or losing, or between qualifying or not qualifying for finals. In the daily training environment, a critical aspect of wellness enhancement is optimising sleep health, which is an essential component of an athlete's recovery and performance [1]. Research has shown that insufficient sleep can impair reaction time, decision-making, and overall physical performance, while also leading to increased risk of injury and pain [2, 3], emphasising its critical role in competitive sports [1]. Yet, among the professionals supporting athletes in the daily training environment, from sport scientists, dietitians, and physiotherapists to psychologists and team physicians, it remains unclear who is best positioned to provide effective sleep guidance. Who has the expertise needed to monitor and interpret sleep data, and who should be delivering sleep-related education sessions and other sleep interventions to athletes? Here, we, a group of individuals from varying backgrounds (sleep scientists, researchers, physicians, sport scientists, sports dietitians, psychologists, and physiotherapists), propose that instead of focusing on any one professional as the definitive solution, a combined approach that draws on the collective expertise of a team of experts is essential. When all professionals work together to deliver a unified, consistent message about sleep, athletes benefit from a more holistic and effective sleep management strategy [4].

## Athletes, Coaches, and Support Staff

Athletes rely on a diverse team of experts to optimise their physical and mental health as well as their performance. Although sleep is a mutual consideration for the many professions that interact with athletes (sport scientists, strength and conditioning coaches, physiotherapists, sports dietitians, sports psychologists, and sports physicians), as discussed in subsequent sections, few, if any, typically possess sufficient formal education or training related to sleep [5–7]. Further, we emphasise that the closeness of any athlete–practitioner relationship depends on individual athlete preferences, the specific training environment, and broader contextual factors. Each sport organisation or team may have very different levels of resourcing, with varying availability of support staff. While some well-resourced teams may include multiple full-time support staff, others may only have part-time specialists, making it difficult to prioritise and coordinate their approach to managing sleep.

### Athletes

Although a multidisciplinary support team is essential, the athlete remains at the core of any sleep management and optimisation strategy. Athletes’ attitudes toward sleep, personal beliefs, and cultural norms may influence their adherence to sleep monitoring and interventions [8–10]. By first understanding the individual athlete, practitioners can identify barriers and facilitators to optimising sleep. This athlete-centred approach allows the multidisciplinary performance support team to tailor education content, adjust environmental variables, and refine sleep strategies in alignment with each athlete’s cultural context and personal motivations. In doing so, the model ensures that interventions are not only evidence-based and coordinated, but also resonate with the athlete’s lived experience, thereby enhancing engagement, adherence, and ultimately, performance outcomes.

### Coaches and Management

Coaches and team management are an important factor in sleep management. As those who often have the most frequent and direct contact with athletes, coaches and managers play a crucial role in identifying and referring athletes who may be experiencing sleep challenges. While they may not always possess knowledge of sleep, their close relationship with athletes allows them to notice behavioural changes, mood disturbances, or signs of fatigue that may indicate poor sleep quantity and/or quality. Beyond identification and referral, coaches and management are key in consulting with the wider team on travel decisions, timing for team meetings, training scheduling, and training availability. They serve as critical links in ensuring that advice from support staff is translated effectively into day-to-day athlete routines. With guidance from sleep specialists and other staff, this may include adjusting training loads after poor sleep, modifying session times based on athlete chronotype, and incorporating short ‘sleep hygiene briefs’ into team meetings. This integrated approach reinforces the holistic model of sleep management that is vital for elite performance [1].

### Sport Scientists and Strength and Conditioning Coaches

Sport scientists, sport physiologists, and strength and conditioning coaches, for example, bring a wealth of knowledge about the body’s response to exercise and the requirements for recovery, emphasising the role of periods of rest in muscle repair and management of training loads [11]. Their advice is often rooted in optimising physical recovery and focuses on how sleep quantity and quality can enhance muscle repair and physiological adaptation. Within a team setting, it is usually their role to implement athlete monitoring systems, often including daily measurement of sleep using either subjective (e.g., daily wellness forms) or objective (e.g., sleep wearables data) forms [12]. Indeed, previous research has reported that athlete monitoring and wellness questionnaires include questions about sleep duration, quality, and disturbances [13], reflecting the understanding that sleep is a key indicator of recovery and readiness. However, most sport scientists are not trained to interpret these data for identification, management, or referral of sleep disorders and more complex sleep issues.

### Physiotherapists

Physiotherapists work closely with athletes to optimise physical health and training/competition availability and are keenly aware of the importance of rest in healing and adaptive processes, as well as the risk of injury associated with poor sleep [8]. Poor sleep quality heightens pain sensitivity and may increase injury risk, while pain‐relief interventions, such as those provided by physiotherapists, may yield improvements in subsequent sleep, indicating a bi-directional relationship between sleep and pain [2, 3]. In this instance, physiotherapists work closely with medical and strength and conditioning colleagues to modify provocative activities during the mechanical functioning of the body, and return to play, rather than on sleep quality itself. Additionally, sports physiotherapists can enhance athlete care by integrating pain education alongside sleep education, fostering a more comprehensive, recovery‐focused approach. In athlete support teams, it is common that physiotherapists travel with athletes and may spend more time with athletes than other members of a support team. If they possess training and expertise in sleep optimisation management, physiotherapists may be a useful resource to help with sleep issues and advice when travelling [5, 6].

### Sports Dietitians

Accredited sports dietitians play a crucial role in optimising dietary habits of sporting teams and athletes. Although their focus is not directly on sleep, their training in complex and integrated health systems can incorporate an athlete’s nutritional needs with a well-rounded sleep strategy to optimise performance and recovery. Food and nutrition can influence sleep onset, depth, and recovery—and likewise sleep health can influence food intake [14, 15]. Food and supplements rich in various nutrients (e.g., tryptophan, omega-3 fatty acids) have been shown to promote restful sleep by enhancing serotonin and melatonin production [16, 17]. Accredited sports dietitians can recommend specific foods to enhance sleep quality and duration, while providing guidance on foods/nutrients that may disrupt sleep (e.g., caffeine). Importantly, they manage these recommendations with a picture of the overall nutrient needs of the individual to support health and performance, a consideration often overlooked by other professionals [18]. Accredited sports dietitians are uniquely placed professionally to provide guidance to athletes to support training and competition nutrition needs, while also navigating the potential impact of nutrition on sleep quality, enabling the factors that promote sleep while managing those which can adversely impact sleep.

### Sports Psychologists

Sports psychologists (and also non-specialised clinical psychologists) understand the importance of sleep for mental recovery, preparation, and performance. They are adept at addressing issues like anxiety and stress that can interfere with sleep. Yet, their focus is more on the psychological components rather than the physiological processes of sleep. Psychologists can provide athletes with techniques to reduce pre-sleep arousal, such as relaxation exercises [19] and other behavioural strategies to help combat insomnia [20]. Of note, psychologists are in the ideal position to administer Cognitive Behavioral Therapy for Insomnia (CBT-I), which is the universally recommended first-line treatment for insomnia in athletes [21]. These methods are particularly effective for athletes experiencing insomnia related to competition anxiety or performance stress. Further, psychologists can guide athletes in developing healthy pre-sleep routines and coping mechanisms to manage negative thought patterns that disrupt sleep. Sport psychologists are often aware of personal struggles of their athletes, both sport and non-sport related, and may have a greater understanding of how these factors may be impacting their sleep [1]. A subset of psychologists are behavioural sleep medicine specialists, who have additional training and knowledge in sleep that can assist athletics programmes [22].

### Sports Physicians

Physicians or medical doctors in the daily team environment (and often while travelling for competition) are typically the go-to professionals for addressing sleep-related problems and disorders. Their comprehensive holistic knowledge mean they can facilitate referrals (e.g., to sleep specialists and physicians) and work with other team members based on what is disrupting sleep. Crucially, they can assess whether an underlying serious condition may be contributing to sleep disturbances. It would also be the team physician’s role to prescribe medications for sleep when appropriate. Physicians, preferably with the assistance of sleep specialists, can diagnose and treat medical issues related to sleep, such as sleep apnea or insomnia, and like other professions have broad responsibilities within a sports team environment that commonly do not allow them to focus deeply on sleep-related issues. However, like many of the aforementioned professions, it is rare for team physicians to have had specialised training in sleep medicine, and they may also receive limited formal education on sleep during their training, unless they have completed formal fellowship training in sleep medicine and are ‘sleep medicine physicians’ [22]. A review study of medical school education across 12 countries reported that, on average, only a few hours—often ranging from 2 to 5—are spent on sleep medicine within their curriculum [7]. While physicians are well equipped to identify and manage medical conditions that impact sleep, their effectiveness in optimising sleep for performance-specific needs may be limited without further specialised training [23] or referrals to others in the wider team.

## Sleep Specialists

Even with access to the full range of multidisciplinary experts, this scattered approach to sleep management may feel fragmented and potentially lead to conflicting advice for athletes without a cohesive strategy. Athletes may need a collaborative framework in which each professional contributes their specialised knowledge to an integrated sleep management plan.

A sleep specialist, whether a qualified sleep scientist, board-certified sleep medicine physician, or board-certified behavioural sleep medicine specialist [22], should be seen as an important part of this collective approach, rather than the sole authority. Typical qualifications for a sleep specialist include advanced degrees such as a PhD in sleep science or related fields (e.g., physiology, psychology, or neuroscience) or medical qualifications with specialisation in sleep medicine. For medical professionals, this often involves board certification in sleep medicine after completing a fellowship programme. Sleep specialists may also possess clinical experience in diagnosing and managing sleep disorders, research experience in areas such as circadian rhythms or sleep architecture, and training in CBT-I or other sleep-specific interventions. Their comprehensive training allows them to assess and measure sleep through polysomnography, actigraphy, and other diagnostic tools, which identify underlying sleep disorders, and tailor interventions accordingly. However, depending on their background and practical experience in sport, they may not fully grasp the nuanced context of high-performance environments, an athletes’ unique physiology, and the challenges and demands placed on them from a psycho-physiological perspective. These individuals are unlikely to have access to the medical records or injury status, which the team physician or physiotherapist has, be aware of the nutritional or psychological challenges of the athlete, nor will they understand the training load history and wellness scores that the sport scientist manages, all of which can impact on the athlete’s sleep and recovery. Additionally, it remains uncommon for teams to employ a sleep specialist in the daily training environment. When they do, it is often in a limited, part-time, or consultancy capacity. This often means that they do not get to know the individual athletes in a day-to-day setting, which may be important for understanding the overall sleep challenges experienced by an individual. Where a sleep specialist is involved, their role should be to work alongside other professionals, ensuring that sleep advice is consistent, science-based, and fully integrated into the broader performance strategy.

### Benefits of a Sleep Specialist

The presence of a sleep specialist, even as a consultant, offers a wide range of benefits within this collaborative model. First, it ensures that the advice given is based on the latest research and best practices specifically tailored to the needs of athletes. Sleep specialists are the most experienced providers to synchronise sleep schedules with training regimens, offer advice on flight selection and jet lag adaptation, optimise sleep environments, facilitate diagnosis and management of clinical sleep disorders, and utilise techniques like napping to maximise performance outcomes. The sleep specialist may also take a lead in upskilling and educating other support staff on matters related to sleep (a ‘train the trainer’ approach). However, they are not the only ones responsible for sleep-related guidance. A successful model relies on effective input and communication among all professionals as outlined above. A sleep specialist can facilitate this communication by serving as a central coordinator who understands the interplay between sleep and various aspects of health and performance. Where more serious and complex sleep disorders are present in athletes, the sleep specialist would likely work closely with the team physician to ensure appropriate treatment plans are in place, including interventions such as continuous positive airway pressure (CPAP), and/or appropriate sleep medications.

### Sleep Health Promotion

Sleep education sessions (either group or individual) are an important component of improving athletes’ understanding and management of sleep and have become commonplace in the elite and professional sport setting [24, 25]. These sessions can be delivered by any of the experts mentioned, depending on the focus of the content, and should include evidence-based, data-driven recommendations to improve sleep hygiene and behaviours [26]. The sleep specialist might be best placed to deliver education as an ‘external voice’ different from the staff in the daily training environment. However, to ensure that the information provided is cohesive and aligns with the athlete’s overall care plan, it is essential that all other relevant experts have the opportunity to provide feedback on the structure and content to be delivered. Additionally, having the team of experts, and ideally coaches, staff, and management present during the sessions with athletes reinforces the unified support system surrounding the athlete, fostering trust and promoting consistency in sleep-related advice [13].

A more comprehensive sleep health promotion programme likely includes elements of screening and routine assessment [13], thoughtful identification of athletes in need of more intensive assessment and treatment for sleep disorders [1], strategies for managing travel fatigue and jet lag when traveling [27], and strategies for tracking/monitoring, optimisation, and improving organisational culture around sleep health [28].

## Sleep Monitoring: Device Performance and Data Considerations

Recent advances in wearables have made continuous sleep monitoring more accessible in sport settings. However, device performance varies considerably depending on the underlying algorithms and the comparison standard [12]. Thus, practitioners should select devices with documented accuracy and reliability [29, 30].

Equally critical is safeguarding athlete sleep data. Sleep metrics may be considered personal health information. Without clear protocols, confidential data may be inadvertently shared among coaches, medical staff, or external service providers [31, 32]. Accordingly, we recommend that each organisation:

1. Obtain explicit, written consent from each athlete specifying which staff roles may access raw sleep data and for what purposes.
2. Store all sleep reports and raw outputs on secure, password‐protected servers or encrypted cloud platforms compliant with local data‐protection legislation.
3. Define tiered access levels (e.g., only performance staff view summary sleep scores; sleep specialists view raw wearable epochs when indicated).
4. Periodically audit data‐sharing practices to ensure compliance and address any breaches promptly.

By selecting validated devices and enforcing clear confidentiality protocols, teams can maximise the benefits of wearable sleep tracking while respecting athlete privacy and maintaining trust [13]. However, it should be noted that wearable sleep technologies come with limitations, and may not be appropriate for every athlete and context. These limitations include the expense (especially in team environments), compliance issues, and the risk of orthosomnia [33, 34].

## Conclusion

Implementing such a model as outlined in this article requires sports organisations to recognise the profound impact of sleep on performance and to foster collaboration among specialists. This necessitates a cultural shift within sports teams, elevating sleep from a general wellness recommendation to a critical component of injury prevention, pain management, and performance optimisation. Evidence from other fields, such as clinical environments, demonstrates the effectiveness of a multidisciplinary approach in improving outcomes—where sleep physicians, behavioural therapists, and other specialists work together to enhance patient care [35]. Similarly, in the field of nutrition, the International Olympic Committee (IOC) emphasises that physique considerations should not be the sole responsibility of one professional but should involve input from a multidisciplinary team, including sports nutritionists, physiologists, psychologists, and medical staff [36]. These parallels reinforce the central proposal of our article: sleep-related decisions and management should be made through expert collaboration rather than individual authority. By fostering teamwork and integrating specialised knowledge, sports teams can ensure a more comprehensive, evidence-based approach to athlete care.

Given that many practitioners mentioned in this article currently lack formal training in sleep, we believe integrating sleep and chronobiology modules into undergraduate and broader educational programmes will ensure future sports professionals are equipped with the knowledge and tools to contribute to the conversation around optimising athlete sleep from their own discipline perspective. We would encourage this consideration in the design of any undergraduate degree programmes that deal with sport, physical activity, and exercise.

In conclusion, as we acknowledge the complexities of athlete health and performance, we must also refine our approach to one of its foundations: sleep. By adopting a collaborative model where sleep specialists work in harmony with other professionals in the daily training environment, we can enhance the quality of sleep management and elevate the overall standard of athlete care. This cooperative approach ensures that all professionals contribute their unique experience and expertise towards a shared goal. This combined strategy promises not only to improve athlete performance but also to revolutionise the way we integrate science, medicine, and sport.
